# Astragaloside IV Suppresses Hepatic Proliferation in Regenerating Rat Liver after 70% Partial Hepatectomy via Down-Regulation of Cell Cycle Pathway and DNA Replication

**DOI:** 10.3390/molecules26102895

**Published:** 2021-05-13

**Authors:** Gyeong-Seok Lee, Hee-Yeon Jeong, Hyeon-Gung Yang, Young-Ran Seo, Eui-Gil Jung, Yong-Seok Lee, Kung-Woo Nam, Wan-Jong Kim

**Affiliations:** 1 Department of Life Science and Biotechnology, College of Natural Sciences, Soonchunhyang University, Asan 31538, Chungcheongnam-do, Korea; ssm4914@naver.com (G.-S.L.); youn6640@naver.com (H.-Y.J.); 94seoyoungran@gmail.com (Y.-R.S.); yslee@sch.ac.kr (Y.-S.L.); kwnam1@sch.ac.kr (K.-W.N.); 2 Soonchunhyang Institute of Medi-bio Science (SIMS), Soonchunhyang University, Cheonan 31151, Chungcheongnam-do, Korea; yhg930205@naver.com; 3 Seoul Center, Korea Basic Science Institute, Seoul 02855, Korea; euigiljung@gmail.com

**Keywords:** astragaloside IV, *Astragalus membranaceus*, huang qi, Astragali Radix, liver, liver regeneration, 70% partial hepatectomy, proliferation, rat

## Abstract

Astragaloside IV (AS-IV) is one of the major bio-active ingredients of huang qi which is the dried root of *Astragalus membranaceus* (a traditional Chinese medicinal plant). The pharmacological effects of AS-IV, including anti-oxidative, anti-cancer, and anti-diabetic effects have been actively studied, however, the effects of AS-IV on liver regeneration have not yet been fully described. Thus, the aim of this study was to explore the effects of AS-IV on regenerating liver after 70% partial hepatectomy (PHx) in rats. Differentially expressed mRNAs, proliferative marker and growth factors were analyzed. AS-IV (10 mg/kg) was administrated orally 2 h before surgery. We found 20 core genes showed effects of AS-IV, many of which were involved with functions related to DNA replication during cell division. AS-IV down-regulates MAPK signaling, PI3/Akt signaling, and cell cycle pathway. Hepatocyte growth factor (HGF) and cyclin D1 expression were also decreased by AS-IV administration. Transforming growth factor β1 (TGFβ1, growth regulation signal) was slightly increased. In short, AS-IV down-regulated proliferative signals and genes related to DNA replication. In conclusion, AS-IV showed anti-proliferative activity in regenerating liver tissue after 70% PHx.

## 1. Introduction

For a long time, medicinal plants have played a key role in pharmacological research studies and drug development. *Astragalus membranaceus* is medicinal plant that has been used in traditional Chinese medicine throughout history; it is also called Astragali Radix. Huang qi (黃芪, in Chinese) is the name for the dried root of *Astragalus membranaceus* and it is mainly produced in China, Mongolia, and Korea [1]. The main components of huang qi are saponins, polysaccharides and flavonoids [2,3,4]. It has been used in traditional Chinese medicine for over 2000 years to treat various diseases including anemia, cardiovascular disorder, weakness, fatigue and fever [4,5]. In recent years, it has been reported that huang qi has anti-oxidative, anti-aging, anti-inflammatory, anti-diabetic, and anti-cancer properties [1,2,3,4].

**Figure 1:**
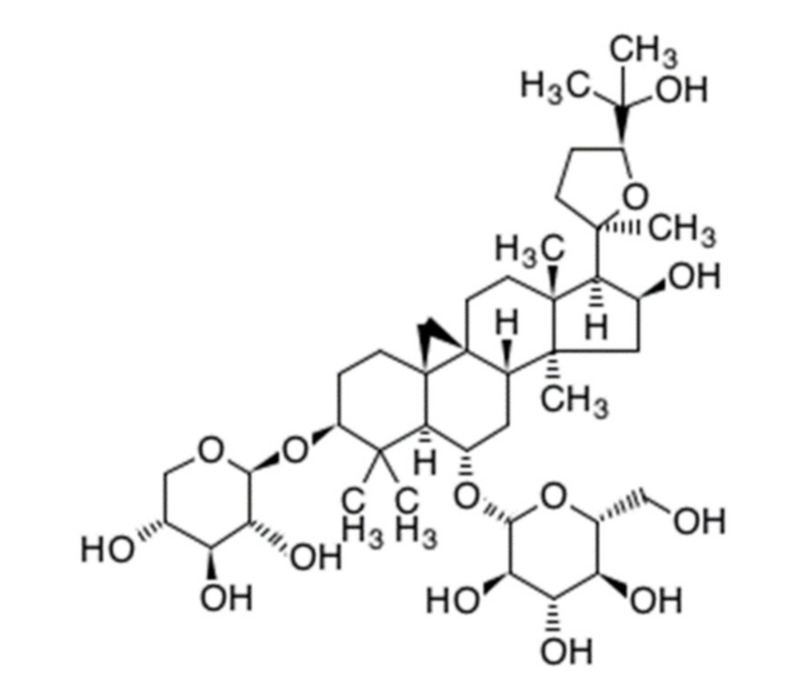
Chemical structure of astragaloside IV. (C_41_H_68_O_14_, molecular weight: 794.97 g/mol).

Astragaloside IV (AS-IV) is a representative bio-active ingredient of huang qi [4,6]. AS-IV is classified as a tetra cyclic triterpenenoid, otherwise known as a protostane which means prototype of steroid (Figure 1) [7]. AS-IV has a similar carbon-tetra-cyclic structure to steroids which have a bio-activities as cholesterol. Recently, it was reported that long-term dietary cholesterol overload negatively affects liver regeneration after PHx. Cholesterol overload reduced hepatic DNA synthesis [8]. While, other steroid, estradiol accelerates liver regeneration [9]. Thus, it was expected that the AS-IV directly has an effects on liver regeneration after PHx. Many reports have shown that AS-IV has anti-oxidative properties and protective effects against hypoxic injury and ischemia-reperfusion injury [10,11,12,13,14,15]. The anti-cancer activities of AS-IV via inhibition of migration and invasion of cancer cells have also been demonstrated [16,17]. Moreover, AS-IV improves diabetic nephropathy, retinopathy, gastropathy, and wound healing [18,19,20,21] Hepato-protective effects against hepatic fibrosis, oxidative stress, and hepatitis B virus were also reported [10,22,23,24,25].

The liver has ability to recover lost functional capacity after chemical or physical injury. After injury, the liver quickly regenerates to meet metabolic demand via the proliferation of hepatic cells. Liver regeneration involves a complex network of growth factors, signaling pathways, and transcriptional factors [26]. Uniquely, liver regeneration occurs without functional loss and through repopulation of mature cells rather than progenitor or stem cells [27].

The 70% partial hepatectomy (PHx) model is a commonly used model to investigate new aspects of liver regeneration [28]. It was first described by Higgins and Anderson in 1931. PHx is a surgical procedure to resect the median and left lateral lobes of the liver, which constitute about 70% of the liver mass [29,30,31]. Liver regeneration is especially rapid in small rodents; full-size restorations have been reported within 7 days in most rodents [27,32]. After PHx, remnant liver tissues undergo three phases. First is priming phase, which is characterized by the stimulation of hepatic mitogen. Hypoxic conditions after PHx and hemo-dynamic factors such as blood pressure are thought to be major stimulators of liver regeneration [33,34,35,36]. The liver is not alone in promoting its own regeneration, and cooperative signals for priming also come from the pancreas, spleen, duodenum, and adrenal glands [37,38]. Second is proliferating phase; DNA replication in hepatocytes started. The last step is growth termination. Transforming growth factor β1 (TGFβ1) is secreted by non-parenchymal cells including hepatic stellate cells, Kupffer cells, and platelets. TGFβ1 plays an important role in ending regeneration through suppression of hepatic proliferation [33,39,40].

The pharmacological effects of AS-IV have been actively studied in recent years and hepato-protective effects of AS-IV have been extensively reported. However, the effects of AS-IV on liver regeneration are not yet fully elucidated. In this study, the effects of AS-IV were investigated in a 70% PHx rat model through measurement of gene expression, the expression of hepatocyte growth factor (HGF, primary hepatic mitogen), and the expression of hepatic proliferation marker protein.

## 2. Results

### 2.1. mRNA Sequencing Analysis

**Figure 2:**
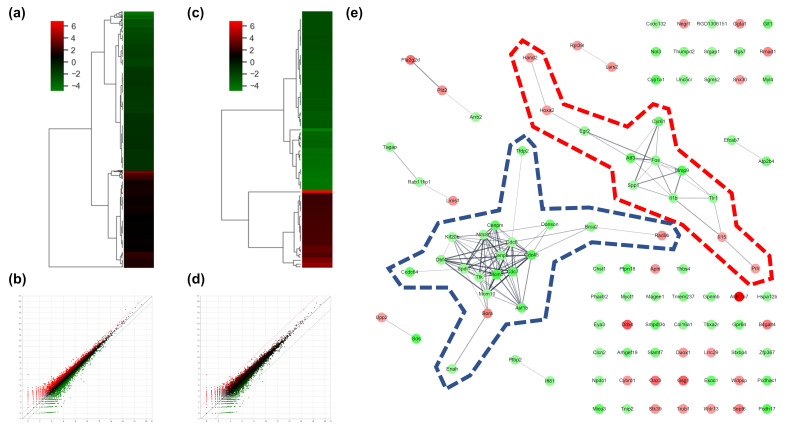
DEGs and gene network. (**a**) heatmap with hierarchical clustering of 2-fold changed DEGs, (**b**) expression pattern for 2-fold changed DEGs, (**c**) heatmap with hierarchical clustering of 5-fold changed DEGs, (**d**) expression pattern for 5-fold changed DEGs, (**e**) Gene network of 5-fold changed DEGs. 20 DEGs (in blue dotted line) and 12 DEGs (in red dotted line) were clustered. Markedly, DEGs within the blue dotted line showed strong relation. 14 other DEGs (not marked) were linked with only one or two genes. The green color in the figure indicates decreased expression; the red color in the figure indicates increased expression.

To determine the effects of AS-IV on the gene expression of regenerating liver tissues after PHx, changes of gene expression were measured 12 h after PHx by mRNA sequencing analysis. The count of differentially expressed genes (DEGs) was 17,048; DEGs exhibiting changes more than 2-fold were considered significant. DEGs exhibiting changes more than 2-fold and 5-fold were 975 and 103 genes, respectively (Figure 2a,b). In the results of the two-fold DEGs, 370 DEGs were up-regulated and 605 DEGs were down-regulated by AS-IV (Figure 2c). Results from the 5-fold DEGs showed that 31 DEGs were up-regulated and 72 DEGs were down-regulated by AS-IV (Figure 2d).

#### 2.1.1. Key Gene Screening and Functional Annotation

To determine the pharmacological effects of AS-IV, we listed key genes from the result and investigated their function through corresponding protein of genes. We listed highly changed DEGs (more than 5-fold) and generated gene networks for the DEGs using a multiple protein search tool within the STRING database; two big cluster networks of DEGs were generated (Figure 2e). One network, which is marked by a blue dotted line in Figure 2e, consisted of 20 DEGs including *Mcm5*, *Cdc45*, *Cdc7*, *Cenpm*, *Asf1b*, *Donson*, *Tfdp2*, *Ccdc64*, *Cenpn*, *Brca2*, *Dbf4*, *Enahm*, *Mcm10*, *Ttk*, *Spdl1*, *Cdc6*, *Ndc80*, *Kif20b*, *Rad9b* and *Bora*. Another big network, marked by the red dotted line of Figure 2e, consisted of 12 genes including *Mmp9*, *Atf3*, *Cyr61*, *Fos*, *Il1b*, *Tlr1*, *Egr2*, *Spp1*, *Il15*, *Prlr*, *Hoxa2*, and *Hand2*. Functional annotation of DEGs in cluster network was conducted using the database for annotation, visualization and integrated discovery (DAVID) bioinformatic tool in three GO (gene ontology) categories including biological process, cellular component, molecular function (Table 1). The 20 DEGs in blue dotted line of Figure 2e were matched with DNA replication initiation, cell division, double-strand break repair via break-induced replication, and DNA duplex unwinding, all of which fall within the biological process category. In the cellular component category, nucleoplasm was matched. In the molecular function category, DNA replication origin binding and chromatin binding were matched. Results from the functional annotation of the DEGs in this cluster (blue dotted line) showed that these genes were related to the DNA replication process of cell division. DEGs in the other clustered network within the red dotted line of Figure 2e were matched with positive regulation of angiogenesis, positive regulation of protein phosphorylation, and positive regulation of apoptotic process. They were matched only the biological process category (Table 1). From the changes in gene expression, strong interaction and function, we considered DEGs within the blue dotted line to be the key DEGs showing the effects of AS-IV.

#### 2.1.2. Pathway Mapping of DEGs 

**Figure 3:**
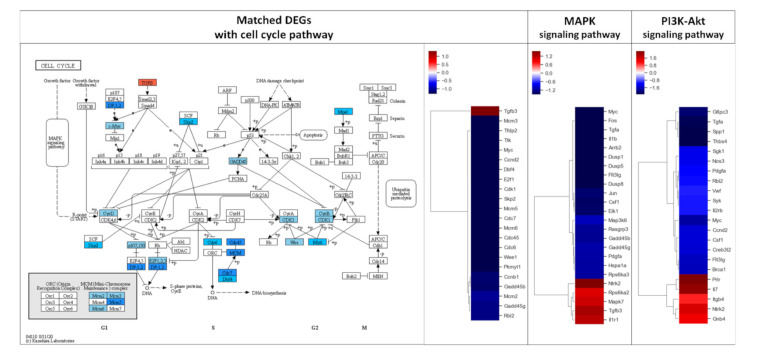
Results of KEGG pathway mapping. 2-fold changed DEGs were analyzed using the KEGG pathway database. 24, 22, and 22 DEGs were included in cell cycle, MAPK signaling, and PI3K-Akt signaling pathways, respectively. AS-IV showed down-regulatory effects on these pathways. Only a few DEGs were up-regulated in these three pathways. MAPK signaling and PI3K-Akt signaling pathways were upstream of the cell cycle pathway. The red and blue color in the figure indicate up-regulation and down-regulation of genes, respectively. Intensity of color is proportional to fold change.

DEGs exhibiting 2-fold changes were further analyzed using the KEGG pathway database to evaluate the effect of AS-IV on signaling in regenerating liver tissues. Remarkably, DEGs were highly matched with three pathways including MAPK signaling, PI3K-Akt signaling, and cell cycle pathways (Table 2). The DEGs in these three pathways showed similar down-regulated patterns; overall, most matched DEGs were down-regulated (Figure 3).

### 2.2. Hepatic Proliferation of Regenerating Liver Tissue

Hepatic proliferation of regenerating liver tissue after AS-IV administration was evaluated via immunohistochemical staining and Western blot analysis for hepatocyte growth factor (HGF) (a mitogen for hepatocytes), cyclin D1 (a marker protein for proliferation), and transforming growth factor β1 (TGF β1) (a terminator for proliferation).

**Figure 4:**
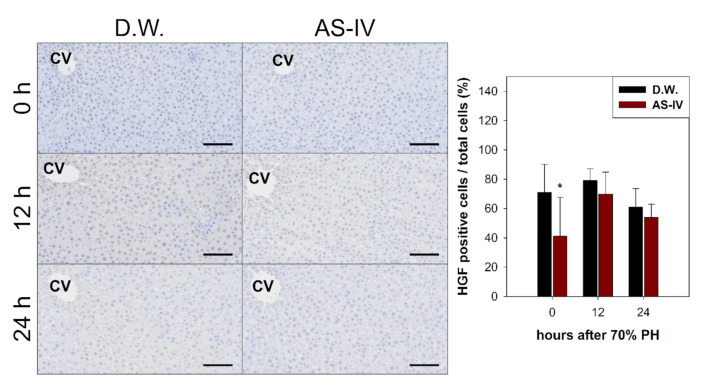
Immunostaining for HGF. HGF-immuno-positive reactions were a brown color after immunostaining. HGF peaked at 12 h after PHx. The AS-IV group showed a low expression of HGF. Hematoxylin was used as a counter stain. (Scale bar indicates 100 μm, CV: central vein, *n* = 3, mean ± standard deviation, *: *p* < 0.05).

**Figure 5:**
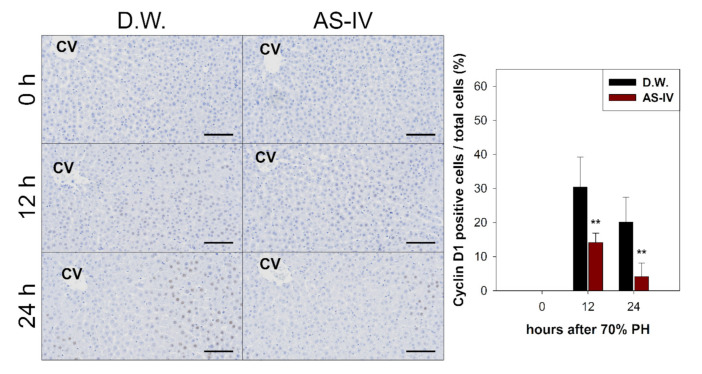
Immunostaining for cyclin D1. Cyclin D1 expression in regenerating liver tissues rapidly increased 12 h after PHx, peaking at 12 h after PHx then decreasing by 24 h. The AS-IV group showed a low expression of cyclin D1. Hematoxylin was used as a counter stain. (Scale bar indicates 100 μm, CV: central vein, *n* = 3, mean ± standard deviation, **: *p* < 0.01).

**Figure 6:**
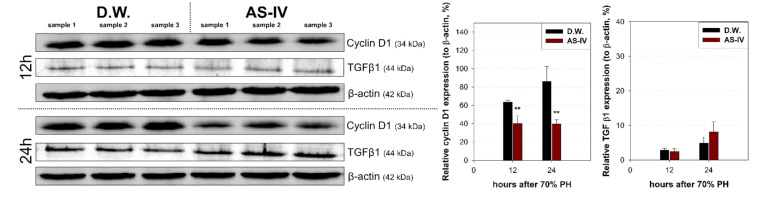
Relative expressions of cyclin D1 and TGF β1. Expression of cyclin D1 and TGFβ1 were analyzed by Western blot analysis. 12 h after PHx, cyclin D1 expression decreased in the experimental group and decreased further by 24 h while showing increased TGFβ1 expression. Relative protein expressions are presented as a ratio of the β-actin loading control. (mean ± standard deviation, *n* = 3, **: *p* < 0.01).

As HGF is a major stimulator of proliferation of hepatocyte during liver regeneration, the expression of HGF was evaluated to determine the hepato-proliferative signal after 70% PHx. Thus, the ratio of HGF positive cells increased immediately after 70% PHx and peaked 12 h after PHx. The ratio of immuno-positive cells in the AS-IV group showed a decrease when compared to controls (Figure 4). Remarkably, immediately after PHx, HGF expression decreased in half of the control group. Cyclin D1 was evaluated to determine the level of proliferation of hepatocyte after proliferative signal. It showed different expression. Cyclin D1 increased since 12 h after PHx with progression for cell cycle. In the AS-IV group, cyclin D1 expression was markedly decreased at 12 and 24 h after PHx (Figure 5). Results of Western blot analysis for cyclin D1 showed a decrease similar to that of the immunohistochemical results in Figure 5 (Figure 6). Cyclin D1 expression was dramatically decreased by AS-IV 24 h after PHx. TGF β1 was also determined by Western blot analysis to be a signal of growth regulation (Figure 6). The expression of TGFβ1 showed no difference 12 h after PHx, however, it was slightly increased with AS-IV treatment 24 h after PHx.

## 3. Discussion

*Aastragalus membranaceus* is one of the oldest known medicinal plants in traditional Chinese medicine. The dried root of this plant is called huang qi and an astragaloside IV (AS-IV) is the core bio-active ingredient of huang qi. Recently, it was reported that AS-IV showed anti-oxidative, anti-cancer, and hepato-protective activity in a variety of experiments [41,42,43]. Looking at recent research, it seems likely that AS-IV has many pharmacological potential. In 2019 Wei et al. reported that AS-IV could improve liver cirrhosis [44]. However, the effects of AS-IV on liver regeneration have not yet been elucidated. The 70% partial hepatectomy (PHx) model has been used in numerous studies of liver regeneration. In this study, we investigated the effects of AS-IV on liver regeneration in a 70% PHx model using mRNA sequencing, immunohistochemistry, and Western blot analysis. Our results suggest that AS-IV could suppress liver regeneration after 70% PHx. After oral administration of AS-IV, many genes changed their expression significantly (Figure 2). To determine the effects of AS-IV, we focused on the functions that are carried out by a group of differently expressed genes (DEGs) rather than the increases or decreases in expression of each individual gene. From the fold change, corresponding protein interactions, and function annotation of DEGs, we found 20 key DEGs including *Mcm5*, *Cdc45*, *Cdc7*, *Cenpm*, *Asf1b*, *Donson*, *Tfdp2*, *Ccdc64*, *Cenpn*, *Brca2*, *Dbf4*, *Enahm*, *Mcm10*, *Ttk*, *Spdl1*, *Cdc6*, *Ndc80*, *Kif20b*, *Rad9b*, and *Bora*, as shown in Figure 2e and Table 1. These genes showed strong functional relationship with cell division and our research suggests that these are the core genes responsible for the function of AS-IV in the regenerating liver. These results provide a potential mechanism for the therapeutic effects of AS-IV. Thus, as it relates to gene expression levels, by suppressing the genes required for molecular binding during DNA replication in the nucleus, AS-IV could inhibit DNA replication during cell division. These results suggest that AS-IV has a potent anti-proliferative effect in the regenerating liver.

DEGs were further analyzed via the KEGG pathway database to investigate the effects of AS-IV on proliferative signaling. Three signaling pathways (MAPK signaling pathway, PI3K-Akt signaling pathway, and cell cycle pathway) were down-regulated by AS-IV (Table 2, Figure 3). Other researchers have also reported similar down-regulation of MAPK signaling by AS-IV [45,46,47]. Decreased hepatocyte growth factor (HGF, hepato-proliferative signal molecule) and cyclin D1 (proliferation marker protein) expression also suggest the same anti-proliferative effects (Figure 4 and Figure 5). Furthermore, AS-IV could affect the growth termination signal as well as could affect proliferative signal. Our extended study results show increased level of transforming growth factor β1 (TGF β1, growth termination signal) in the liver 24 h after PHx (Figure 6). In summary, AS-IV was revealed to have anti-proliferative effects in the regenerating liver via changes in gene expressions and protein expressions related to cell division and proliferative signals. 

And So, AS-IV showed anti-proliferative effects in regenerating liver tissues. Thus, for the purpose of tissue regeneration through encouraging normal cell division, it is likely that AS-IV is unsuitable for liver regeneration. However, AS-IV could be applied for other purposes, such as for reducing oxidative injury after PHx surgery. After PHx, the liver is rapidly regenerated. In parallel, oxidative stress also rapidly increased. Some reports have shown that AS-IV has anti-oxidative and hepato-protective activities [48,49]. Additionally, our results could be used as basic study data for the anti-cancer activities of AS-IV with other studies. In recent, similar anti-cancer effects of AS-IV were reported including blocking of MAPK signal, decreased PCNA and Ki67 expression, and trigging G1 arrest in tumor cell [45,50].

## 4. Materials and Methods

### 4.1. Animals and Experimental Design

Male rats (SD strain, 8 weeks) were obtained from DBL Co., Ltd. (Eumseong, Korea). They were housed in an environmentally controlled room at 25 ± 1 °C, 12/12-light/dark cycle, and relative humidity 60 ± 5% with free access to standard food pellets and water (ad libitum). All animal experiments and procedures were performed in accordance with the guidelines for the care and use of laboratory animals of the national institutes of health (NIH) and after approval by the institutional animal care and use committee (IACUC) at the Sooonchunhyang University (permission No.: SCH20-0002).

**Figure 7:**
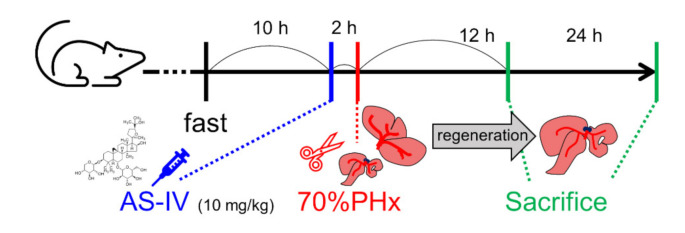
Administration of AS-IV and 70% PHx. Rats were received 10mg/kg of AS-IV diluted in D.W. (experimental group) or D.W. (control group) 2 h before 70% PHx and were sacrificed at 12 h or 24 h after PHx.

Rats were randomly divided into control and experimental groups depending on treatment and sacrifice time. Both groups consisted of six rats. Three rats of each group were sacrificed 12 h after PHx and the other three rats were sacrificed 24 h after PHx. The rats in the experimental group received intragastric administration of astragaloside IV (AS-IV, 10 mg/kg) [4,13,19,22,51]. AS-IV was diluted in 1.5 mL of D.W. [52]. AS-IV (product No. #A3305, purity > 98.0%, HPLC) was purchased from the Tokyo Chemical Industry Co., Ltd. (Tokyo, Japan). The rats in the control group received the same volume of D.W. by intragastric administration. AS-IV and D.W. were administrated 2 h before surgery (Figure 7) [53,54,55,56,57,58].

To establish the liver regeneration model, a 70% partial hepatectomy (PHx) involving resection of the median and left lateral lobes was performed under anesthesia as previously described by Higgins and Anderson [29]. Animals were fasted for 12 h before surgery. Rats were sacrificed at 12 or 24 h after 70% PHx [39]. Regenerated remnant liver tissue was collected for analyses.

### 4.2. RNA Sequencing Analysis

TRIzol^®^ reagent (Invitrogen, Carlsbad, CA, USA) was used for isolation of total RNA from liver tissue [59]. cDNA libraries were generated and purified using QuantSeq 3′ mRNA-Seq Library Prep Kit for Illumina (LEXOGEN, Vienna, Austria) according to manufacturer’s instructions. High-throughput sequencing was performed as single-end 75 base pair sequencing using a NextSeq 500 (Illumina, Inc., San Diego, CA, USA). Raw reads were processed by BBDuk and aligned to the reference genome (rat, rn6, UCSC) using Bowtie2 [60]. The alignment file was assembled and estimated their abundances. Differentially expressed genes (DEGs) were determined based on counts from unique and multiple alignments with coverage in the Bedtools. The read count data were processed based on a quantile normalization method using EdgeR within R [61]. Genes were classified based on the database for annotation, visualization and integrated discovery (DAVID) and Medline databases (http://david.abcc.ncifcrf.gov/, accessed on 8 November 2020). DEGs exhibiting changes more than 2-fold were considered significant [59]. DEGs were also analyzed via the Kyoto Encyclopedia of Genes and Genomes (KEGG) mapper (address: https://www.genome.jp/kegg/tool/map_pathway2.html, accessed on 8 November 2020). DEGs were also analyzed based on protein-protein interactions via multiple protein searching tool within STRING database (ver. 11.0, address: http://string-db.org/db.org/, accessed on 8 November 2020).

### 4.3. Immunohistochemistry

Liver tissue was removed and immediately fixed in 10% neutral buffered formalin. It was then embedded in paraffin according to the routine process for light microscopy. The paraffin block was then cut into 4 μm thicknesses by rotary microtome (RM2235, Leica Biosystems, Germany). Antigen retrieval was conducted with sodium citrate buffer (10 mM, pH 6.0) at 95 °C. Heated sections were cooled for 30 min at room temperature. Sections were then incubated in 3% H_2_O_2_ (#1146, DUCSAN PURE CHEMICALS, Korea) and incubated in 5% bovine serum albumin (BSA, A7906, Merck KGaA, Darmstadt, Germany). Primary antibodies against Cyclin D1 (ab134175, Abcam plc., UK) and HGF (ab83760, Abcam plc., UK) were added. An HRP-conjugated secondary antibody (Thermo fisher scientific, Waltham, MA, USA) was then added. Immuno-detection was conducted with 3,3′-diaminobenzidine (SIGMAFAST™, Merck KGaA, Darmstadt, Germany). Counter staining was conducted using hematoxylin. All procedures were carried out in a humidified chamber to prevent the drying out of tissues. Tissues were observed using a microscope (CKX53, Olympus, Tokyo, Japan). Counts of positive reacted cells and total cells were measured by the color deconvolution tool within TMARKER (Ver. 2.146, open-source software) [62,63].

### 4.4. Western Blot Analysis

Total protein was extracted from liver tissues using protein extraction solution (PRO-PREP™, iNtRON BIOTECHNOLOGY, Seongnam, Korea) on ice and was determined by bicinchoninate (BCA) calorimetric assay kit (#23227, Thermo fisher scientific, USA) according to manufacturer’s instruction. The protein was separated by 12% SDS-PAGE and transferred to PVDF (IPVH00010, Merck KGaA, Germany). The PVDF was then blocked with 5% skim milk (#232100, BD, Franklin Lakes, NJ, USA) and incubated with a primary antibody diluted in 0.5% skim milk overnight at 4 °C. The primary antibody against β-actin (A5316, Merck KGaA, Darmstadt, Germany) was diluted to 1:6000. Primary antibodies against cyclin D1 (ab134175) and TGF β1 (ab92486) were purchased from Abcam plc. (Cambridge, UK) and diluted to 1:3000. The membrane was further incubated with an HRP-conjugated secondary antibody (Thermo fisher scientific, USA) at room temperature for 1 h, followed by washing with PBS. Protein expression was detected by enhanced chemiluminescence (ECL) solution (K-12049-D50, Advansta Inc., San Jose, CA, USA) and chemiluminescence imaging system (GBox ichemi XL, Syngene, UK). β-actin was used as loading control. The relative expression level of target protein was expressed as ratio of β-actin [64].

### 4.5. Statistical Analysis

All quantitative data are presented as mean ± standard deviation (SD) from three independent experiments. Statistical analyses were performed using IBM SPSS statistics for windows (ver. 25, IBM, New York, NY, USA). Student’s *t*-tests were used to analyze differences between control and the experimental group. Data with a *p*-value less than 0.05 were considered statistically significant. (*: *p*-value < 0.05, **: *p*-value < 0.01)

## 5. Conclusions

**Figure 8:**
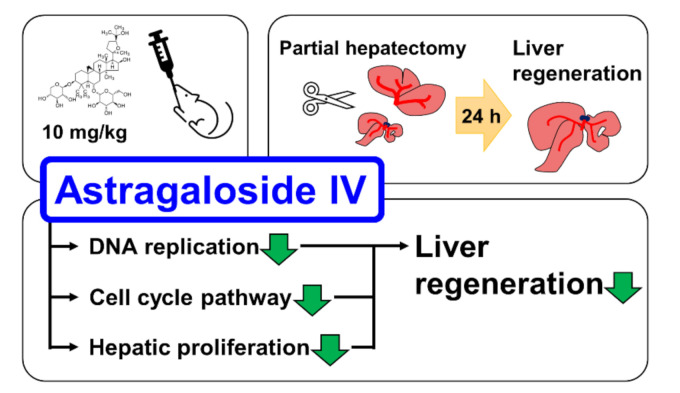
Graphical abstract of study. AS-IV suppressed hepatic proliferation of regenerating liver tissues after 70% PHx.

Astragaloside IV (AS-IV) is the major bio-active component of huang qi (the dried root of *Astragalus membranaceus*, a traditional Chinese medicinal plante). In present study, we demonstrated the pharmacological effects of AS-IV on regenerating rat liver tissue after 70% partial hepatectomy. AS-IV down-regulated proliferative signals, genes related to DNA replication, and cyclin D1 expression (Figure 8). In conclusion, AS-IV showed anti-proliferative activities in regenerating liver tissue.

## Figures and Tables

**Table 1:** Functional annotation of clustered DEGs by DAVID in Figure 2e.

Gene network		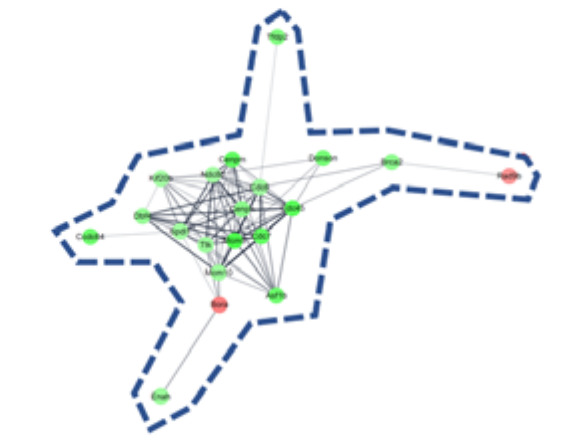	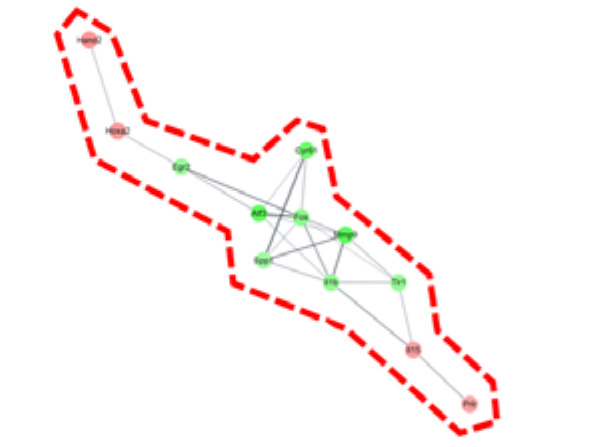
Clustered genes		*Mcm5*, *Cdc45*, *Cdc7*, *Cenpm*, *Asf1b*, *Donson*, *Tfdp2*, *Ccdc64*, *Cenpn*, *Brca2*, *Dbf4*, *Enahm*, *Mcm10*, *Ttk*, *Spdl1*, *Cdc6*, *Ndc80*, *Kif20b*, *Rad9b*, *Bora*	*Mmp9*, *Atf3*, *Cyr61*, *Fos*, *Il1b*, *Tlr1*, *Egr2*, *Spp1*, *Il15*, *Prlr*, *Hoxa2*, *Hand2*
GO category	Biological process	- DNA replication initiation - Cell division - Double-strand break repair via break-induced replication - DNA duplex unwinding	- Positive regulation of angiogenesis - Positive regulation of protein phosphorylation - Positive regulation of apoptotic process
	Cellular component	- Nucleoplasm	(not matched)
	Molecular function	- DNA replication origin binding - Chromatin binding	(not matched)

**Table 2:** Results of pathway mapping for 2-fold changed DEGs by AS-IV.

KEGG Pathway	Count of Genes		
	Matched	Up-Regulated	Down-Regulated
MAPK signaling pathway	24	5	19
PI3K-Akt signaling pathway	22	5	17
Cell cycle pathway	22	1	21

## Data Availability

We want to exclude this statement. Our study did not report public dataset.

## Sample Availability

Astragaloside IV is available from the authors or Tokyo Chemical Industry Co., Ltd. (Tokyo, Japan). Product No. #3305.
